# Microbial dysbiosis-associated NMDAR hyperfunction in post-infectious irritable bowel syndrome with visceral hypersensitivity

**DOI:** 10.1093/gastro/goaf065

**Published:** 2025-11-24

**Authors:** Lijun Du, Fangli Cheng, Weida Shen, Yubin Zhu, Chenhong Lin, Zhenzhen Fan, Ning Dai

**Affiliations:** Department of Gastroenterology, Sir Run Run Shaw Hospital, School of Medicine, Zhejiang University, Hangzhou, Zhejiang, P. R. China; Department of Diagnostic Ultrasound and Echocardiography, Sir Run Run Shaw Hospital, School of Medicine, Zhejiang University, Hangzhou, Zhejiang, P. R. China; Key Laboratory of Novel Targets and Drug Study for Neural Repair of Zhejiang Province, School of Medicine, Hangzhou City University, Hangzhou, Zhejiang, P. R. China; Department of Gastroenterology, Sir Run Run Shaw Hospital, School of Medicine, Zhejiang University, Hangzhou, Zhejiang, P. R. China; Endoscopic Center, Sir Run Run Shaw Hospital, School of Medicine, Zhejiang University, Hangzhou, Zhejiang, P. R. China; Department of Gastroenterology, Sir Run Run Shaw Hospital, School of Medicine, Zhejiang University, Hangzhou, Zhejiang, P. R. China; Department of Gastroenterology, Sir Run Run Shaw Hospital, School of Medicine, Zhejiang University, Hangzhou, Zhejiang, P. R. China

**Keywords:** irritable bowel syndrome, visceral hypersensitivity, *N*-methyl-D-aspartate receptor, insula, hippocampus

## Abstract

**Background:**

Irritable bowel syndrome (IBS) is a condition with undetermined pathophysiology. Dysregulation of the gut–brain axis has been implicated in its development. This study aimed to investigate the association between gut microbial dysbiosis and brain *N*-methyl-D-aspartate receptor (NMDAR) hyperfunction in a mouse model of post-infectious IBS (PI-IBS) with visceral hypersensitivity.

**Methods:**

Four-week-old mice were divided into four groups: control mice, PI-IBS mice, PI-IBS mice co-housing with normal mice, and PI-IBS mice administrated with a cocktail of antibiotics. The PI-IBS mouse model was established by using *Trichinella spiralis* infection. Visceral sensitivity was measured by using the abdominal withdrawal reflex in response to colorectal distension. c-FOS and NMDAR expression in the insula, hippocampus, and hypothalamus were evaluated by using immunostaining and Western blot, respectively. Additionally, NMDAR-mediated evoked excitatory postsynaptic currents (eEPSCs) in these brain regions were recorded by using patch clamp techniques.

**Results:**

Visceral hypersensitivity was observed in PI-IBS mice compared with control mice. Increased c-FOS expressions were observed in the insula and hippocampus of PI-IBS mice. Although no changes in the NMDAR subtypes expression were observed, enhanced NMDAR-mediated eEPSCs were detected in the insula and hippocampus of PI-IBS mice compared with control mice. Co-housing and antibiotics treatment effectively reduced NMDAR-mediated eEPSCs and alleviated visceral hypersensitivity.

**Conclusion:**

Gut microbiota dysbiosis may serve as an initiating factor for NMDAR hyperfunction in PI-IBS with visceral hypersensitivity.

## Introduction

Irritable bowel syndrome (IBS) is a common symptom-based disorder that is characterized by the presence of abdominal pain or discomfort, affecting ∼7%–15% of the general population [1, 2]. The pathogenesis of IBS involves multiple factors, including visceral hypersensitivity, microbial dysbiosis, and psychological distress [3–7]. Gut–brain bidirectional interactions have been implicated in the development of IBS [8]. Clinical studies have identified the insula, hippocampus, and hypothalamus as critical components of the functional circuit underlying IBS [9]. Furthermore, accumulating evidence suggests that gut bacteria can influence the nervous system through various mechanisms, consequently impacting host physiology [10].

Gut–brain communication is mediated by ionotropic glutamate receptors, such as the α-amino-3-hydroxy-5-methyl-4-isoxazole propionic acid receptor (AMPAR) and *N*-methyl-D-aspartate receptor (NMDAR). These receptors are widely expressed in the nervous system and play critical roles in excitatory synaptic transmission [11]. Previous study has revealed that NMDARs are abundantly expressed in colonic mucosal cells and are involved in visceral hypersensitivity [12]. Our previous work demonstrated gut microbiota dysbiosis and elevated NMDAR expression in the enteric nervous system of post-infectious IBS (PI-IBS) mice with visceral hypersensitivity [13]. Furthermore, peripheral nociceptive stimuli have been shown to activate spinal NMDARs, enhancing neuronal excitation in the dorsal horn and facilitating nociceptive transmission [14, 15]. In the present study, we employed co-housing and antibiotics treatments to induce gut microbiota remodeling and disruption, aiming to investigate the role of gut dysbiosis-related NMDAR activation in the central nervous system during the maintenance of visceral hypersensitivity by using a PI-IBS mouse model.

## Methods

### PI-IBS mouse model establishment

Experiments were performed by using male Swiss NIH mice (Guangdong Medical Laboratory Animal Center, Guangzhou, China). Four-week-old mice were housed under controlled environmental conditions and provided with free access to food and water. Thirty-two mice were randomly divided into four groups: control group, PI-IBS group, PI-IBS mice co-housing with normal mice (CO-PI-IBS), and PI-IBS mice administered with a cocktail of antibiotics (AB-PI-IBS) [13]. The PI-IBS model was induced by the intragastric inoculation of 300–400 *Trichinella spiralis* larvae in 0.1 mL normal saline, which resulted in visceral hypersensitivity without histological signs of inflammation, as previously described [16]. Continuous short-term co-housing was employed as the preferred strategy in studying intestinal diseases, in order to effectively control changes attributed to microbial factors [17]. For antibiotics treatment, mice were intragastrically administered 200 mg/kg of neomycin sulfate (Cat #MB1716; Meilune, Dalian, China), 100 mg/kg of vancomycin (Cat #MB1260; Meilune), 200 mg/kg of metronidazole (Cat #MB2200; Meilune), and 200 mg/kg of ampicillin (Cat #MB1378; Meilune) once daily for a duration of 4 weeks [18]. All experimental procedures were conducted in accordance with the guidelines of the Animal Care and Use Committee of Zhejiang University (No.11898).

### Abdominal withdrawal reflex assessment

Visceral sensitivity was assessed by the measuring abdominal withdrawal reflex (AWR) arising in response to colorectal distension at baseline and 8 weeks post-infection [19]. Colorectal distension was performed in ascending order at pressures of 20, 40, 60, and 80 mmHg, following a previous description [20]. The response to colorectal distension was quantified by using an AWR scoring system: 0, no behavioral response; 1, brief head movement followed by immobility; 2, contraction of abdominal muscles; 3, lifting of abdomen; and 4, body flexion and pelvic lift [21, 22]. The threshold intensity was determined by gradually increasing the colorectal distension pressure (up to 100 mmHg) until a stimulus value evoked lifting of the abdomen and body arching [22, 23]. Each measurement was conducted three times and independently recorded by two observers to ensure methodological rigor.

### Immunohistochemistry

Immunohistochemistry stains were utilized to visualize the c-FOS distribution in specific regions of the brain, including the insula, hippocampus, and hypothalamus. The detection of c-FOS served as a proxy for assessing neuronal activity. After being deparaffinized and hydrated, the sections were treated with 0.3% hydrogen peroxide to block the activity of the endogenous peroxidase, followed by heat-mediated antigen retrieval. Then, the primary antibody against c-FOS (1:500; Cat #ab208942; Abcam, Cambridge, UK) was applied to the sections and incubated for 1 h. Afterwards, the sections were treated with horseradish peroxidase (HRP)-labeled goat anti-mouse IgG (Cat #DS0003; ZSGB, Beijing, China) for 30 min. Immunohistochemical positivity was determined by using diaminobenzidine (DAB kit; Cat #ZLI9019; ZSGB) staining. c-FOS expression was visualized and quantified by using Image Pro Plus 6.0.

### Western blot

Total protein extraction from the insula, hippocampus, and hypothalamus was performed by using radio immunoprecipitation assay (RIPA) lysis buffer supplemented with a protease inhibitor cocktail and protein quantification was determined by using the BCA Protein Assay kit (Cat #CW0014; CWBIO, Beijing, China). Protein samples were separated through sodium dodecyl sulfate-polyacrylamide gel electrophoresis (SDS-PAGE) gel electrophoresis and then transferred onto polyvinylidene fluoride (PVDF) membranes (0.20-μm pore; Millipore, Massachusetts, USA). The membranes were blocked with 5% skimmed milk and then probed overnight at 4°C with primary antibodies against NMDAR1 (1:1,000; Cat #ab109182; Abcam), NMDAR2A (1:500; Cat #ab124913; Abcam), NMDAR2B (1:500; Cat #ab93610; Abcam), and β-actin (1:1,000; Cat #CW0096; CWBIO). Following primary antibody incubation, the membranes were further incubated with corresponding HRP-conjugated secondary antibodies (1:3,000; Cat #CW0103; CWBIO) at room temperature for 2 h. The protein bands were visualized by using a luminescent image analyser (LAS 4000; FUJIFILM, Tokyo, Japan) and the immunoblot signals were quantified by using Image J software.

### Acute slice preparation

Acute brain slices were prepared by following established procedures [24]. Mice were anaesthetized with sodium pentobarbital (50 mg/kg) for decapitation. The brains were rapidly excised and submerged in a protective solution with the following composition (in mM): 235 sucrose, 20 glucose, 2.5 KCl, 0.5 CaCl_2_, 1.25 NaH_2_PO_4_, 26 NaHCO_3_, 7 MgCl_2_, and 2 pyruvate (pH 7.3, 310 mOsm). The protective solution was pre-chilled to ∼4°C and bubbled with 95% O_2_ and 5% CO_2_. A vibrating slicer (VT1200S; Leica Biosystems, Nussloch, Germany) was used to obtain coronal slices (300 μm) from the insula, hippocampus, and hypothalamus. The slices were initially incubated at 32°C for 60 min in regular artificial cerebrospinal fluid (pH 7.4, 310 mOsm) that contained the following substances (in mM): 10 glucose, 1.25 NaH_2_PO_4_, 26 NaHCO_3_, 126 NaCl, 2.5 KCl, 1 sodium pyruvate, 2 CaCl_2_, and 1 MgCl_2_. Following the incubation period, the slices were kept at room temperature for 0.5–5 h.

### Patch clamp recording

The brain slices were transferred to a submersion-type recording chamber for electrophysiological recordings and continuously perfused with oxygenated artificial cerebrospinal fluid (95% O_2_ and 5% CO_2_) at 3 mL/min. Patch clamp recordings were performed on neuronal cells by using an upright microscope (BX51WI; Olympus, Tokyo, Japan) equipped with infrared differential interference contrast. To selectively block gamma-aminobutyric acid subtype A receptor-mediated currents, 100 mM of picrotoxin (Cat #HY101391; MCE, Shanghai, China) was added to the artificial cerebrospinal fluid during all cell recordings at 32°C. Prior to recording, cells were voltage clamped at –70 mV for ≥10 min prior to establishing a stable baseline, during which 20–30 evoked excitatory postsynaptic currents (eEPSCs) were continuously and reliably recorded and averaged. At this stage, the recorded currents were considered to be mediated by AMPAR, as the presence of Mg^2+^ blocked the NMDAR channels. Then, cells were voltage clamped at +50 mV and steady 20–30 eEPSCs were continuously recorded and averaged. By removing Mg^2+^ during depolarization, the NMDAR channels opened and contributed to the mixed currents from both the AMPAR and NMDAR channels. Based on the distinct dynamic characteristics of the AMPAR and NMDAR channels (AMPAR channels open and close quickly, with a short duration, while NMDAR channels are slow channels, with a long duration), the currents measured at 50 ms after the generation of mixed currents were primarily mediated by NMDARs. The ratio of NMDAR to AMPAR currents, referred as the NMDAR tone, was calculated. During the recording, changes in membrane resistance and leakage currents were monitored. Data with leak currents bigger than –200 pA and series resistances that changed by <20% were considered acceptable. For whole-cell recordings, glass electrodes with resistances of 5–6 MΩ were filled with (in mM): 125 CsMeSO_3_, 10 HEPES, 10 EGTA, 4 ATP-Na_2_, 0.4 GTP-Na, 5 4-AP, 8 TEA-Cl, 1 MgCl_2_, and 1 CaCl_2_ (pH 7.3–7.4, 280–290 mOsm). Synaptic responses in the recorded cells were evoked by using orthodromic stimulation, with the intensity of the stimulus adjusted to obtain a stable response in the cells. Evoked synaptic currents were stimulated every 8 s. Neuronal recordings were obtained by using Axopatch 700B amplifiers (Molecular Devices, San Jose, CA, USA) and digitized by using pClamp 10.6 software (Molecular Devices).

### Statistical analysis

Statistical analyses were conducted by using GraphPad Prism 8.0 (San Diego, CA, USA) and SPSS 25 (Chicago, IL, USA). Intergroup comparisons were analysed by using the Student’s *t*-test (parametric data) or Mann–Whitney *U* test (non-parametric data), while multigroup comparisons were assessed by using one-way analysis of variance (ANOVA). Effect sizes for between-group differences were quantified by using Hedges’ g with 95% confidence interval (CI). Statistically significant differences were defined as a *P*-value of <0.05.

## Results

### PI-IBS model establishment

**Figure 1.**
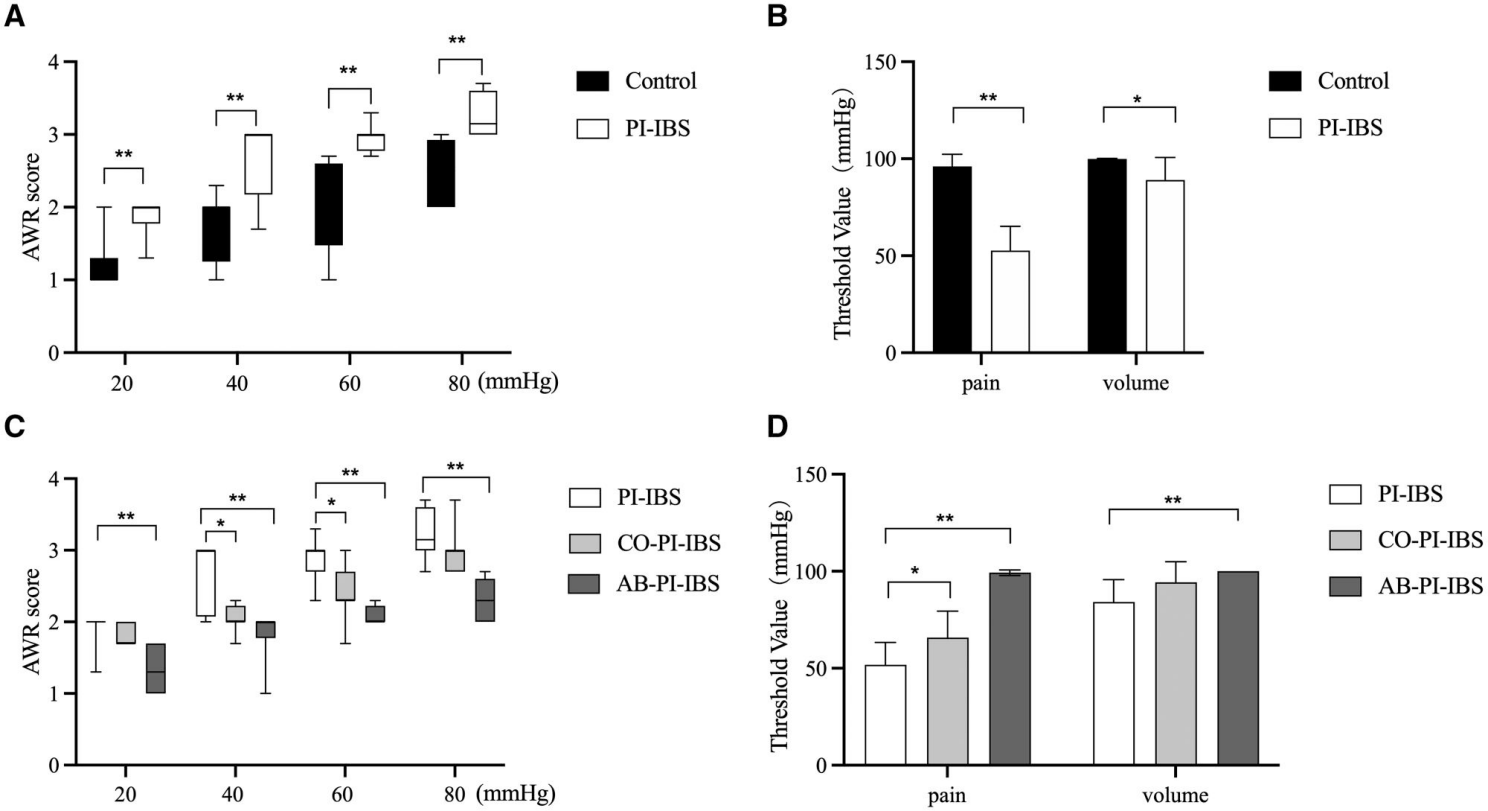
AWR to colorectal distension. (A, B) Higher AWR scores at all levels of distension, lower pain threshold, and volume threshold in PI-IBS mice compared with control mice. (C, D) Lower AWR score at 40 and 60 mmHg of distension, higher pain threshold in CO-PI-IBS mice compared with PI-IBS mice. Lower AWR scores at all levels of distension, higher pain threshold, and volume threshold in AB-PI-IBS mice compared with PI-IBS mice. Pain threshold: the lowest pressure elicited lifting of abdomen. Volume threshold: the lowest pressure elicited body arching. *P < 0.05; **P < 0.01.

At 8 weeks after inoculation, PI-IBS mice exhibited elevated AWR scores at all levels of distension compared with control mice (all *P *< 0.01). Additionally, PI-IBS mice demonstrated a reduced pain threshold (*P *< 0.01) and volume threshold (*P *< 0.05) than did control mice. After co-housing with normal mice, CO-PI-IBS mice showed decreased AWR scores at 40 and 60 mmHg of colorectal distension (*P *< 0.05), along with an increased pain threshold (*P *< 0.05) compared with PI-IBS mice. Furthermore, antibiotics treatment led to lowered AWR scores at all levels of distension (all *P *< 0.01) and increased pain threshold (*P *< 0.01) and volume threshold (*P *< 0.01) in AB-PI-IBS mice when compared with PI-IBS mice (Figure 1).

Histological analysis revealed no significant differences in the ileal and colonic inflammation scores among the four groups (control, PI-IBS, CO-PI-IBS, and AB-PI-IBS) at 8 weeks, indicating the resolution of inflammation.

### c-FOS and NMDARs expression evaluation

**Figure 2.**
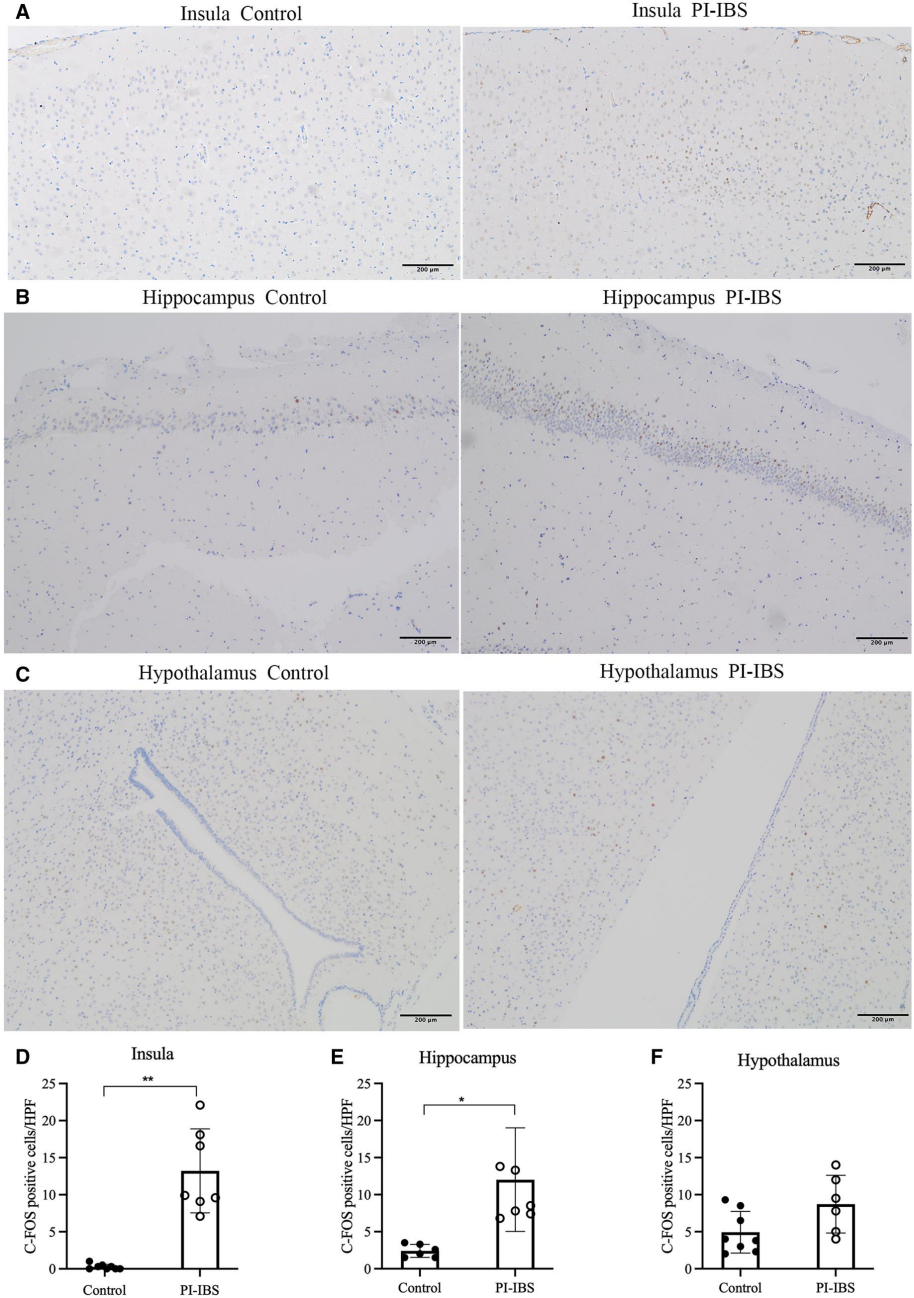
c-FOS expression in PI-IBS and control mice. (A, D) Increased c-FOS expression in the insula of PI-IBS compared with control mice. (B, E) Increased c-FOS expression in the hippocampus of PI-IBS compared with control mice. (C, F) No difference in c-FOS expression in the hypothalamus between PI-IBS and control mice. *P < 0.05; **P < 0.01.

**Figure 3.**
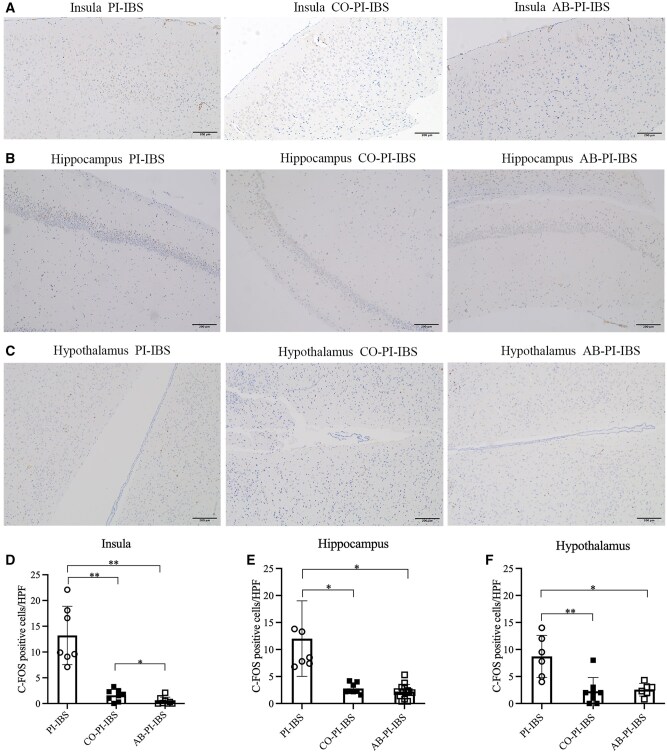
c-FOS expression in PI-IBS mice with co-housing and antibiotics treatment. (A, D) Decreased c-FOS expression in the insula of PI-IBS mice after co-housing and antibiotics treatment. (B, E) Decreased c-FOS expression in the hippocampus of PI-IBS mice after co-housing and antibiotics treatment. (C, F) Decreased c-FOS expression in the hypothalamus of PI-IBS mice after co-housing and antibiotics treatment. *P < 0.05; **P < 0.01.

In control mice, the expression of c-FOS was observed scattered throughout the insula, hippocampus, and hypothalamus. In PI-IBS mice, a significant increase in c-FOS expression was found in the insula (*P *= 0.001, Hedges’ g = 3.359, 95% CI 1.786–4.931) and hippocampus (*P *= 0.001, Hedges’ g = 1.849, 95% CI 0.548–3.151), while no significant difference was observed in the hypothalamus (*P *= 0.056, Hedges’ g = 2.209, 95% CI 0.773–3.644) when compared with control mice (Figure 2). Following co-housing and antibiotics treatment, there was a significant decrease in c-FOS expression in the insula, hippocampus, and hypothalamus (all *P *< 0.05, Figure 3).

**Figure 4.**
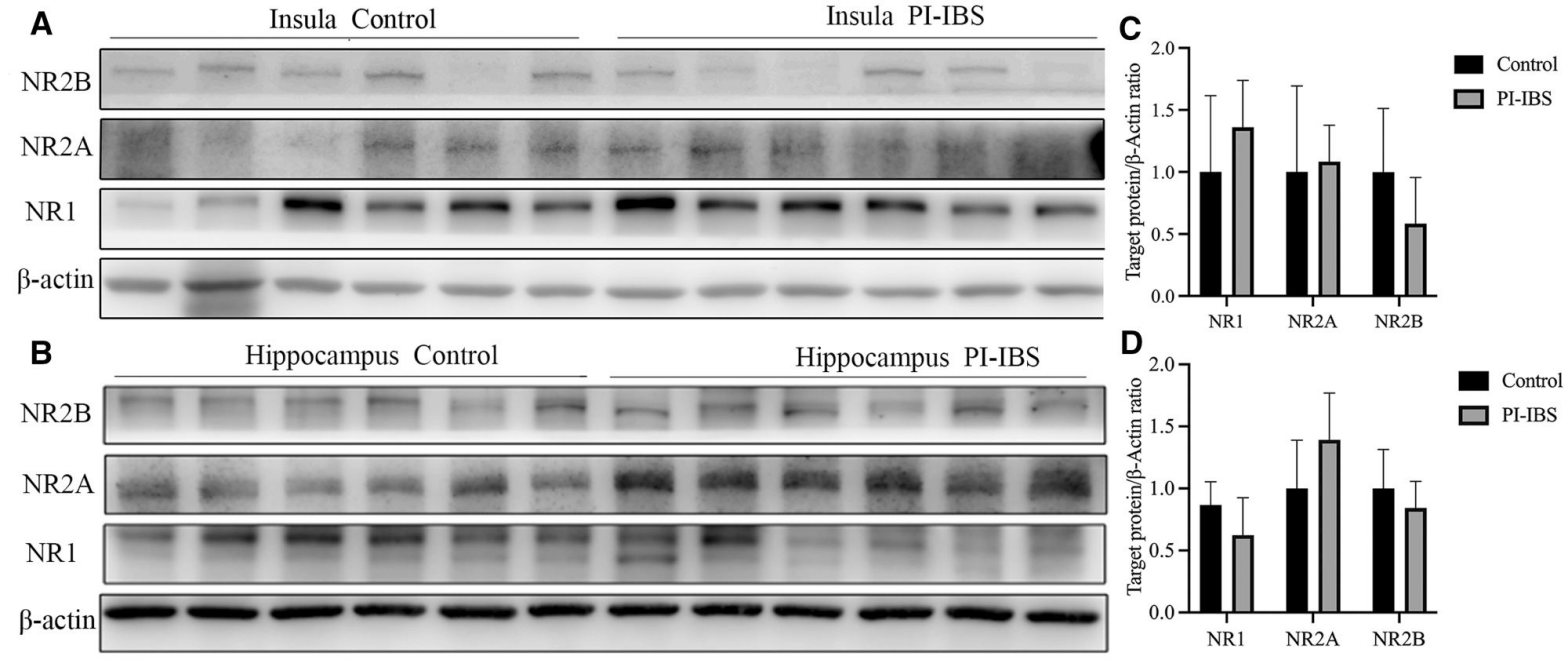
NMDAR expression in PI-IBS and control mice. (A, C) No difference in NMDAR expression in the insula between PI-IBS and control mice. (B, D) No difference in NMDAR expression in the hippocampus between PI-IBS and control mice.

**Figure 5.**
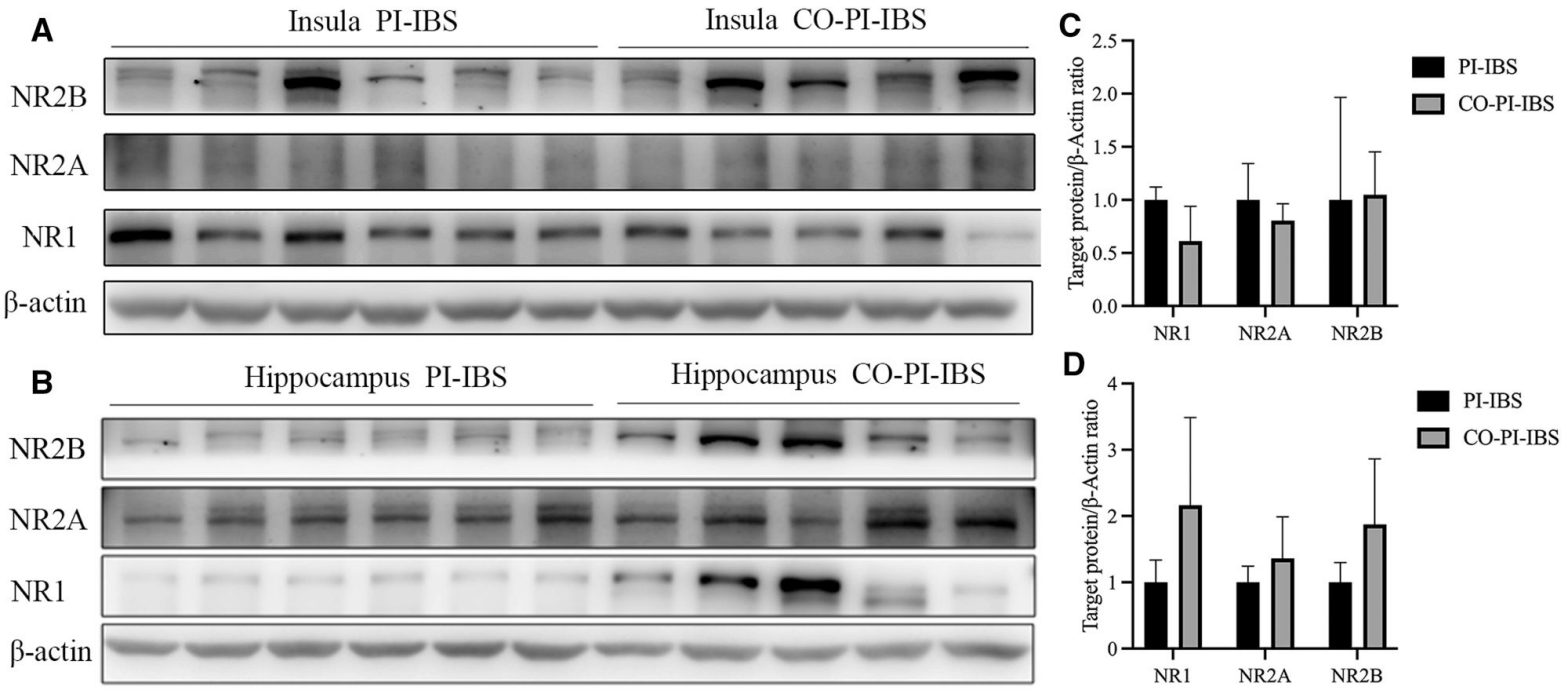
NMDAR expression in PI-IBS mice with co-housing treatment. (A, C) No difference in NMDAR expression in the insula of PI-IBS mice with co-housing treatment. (B, D) No difference in NMDAR expression in the hippocampus of PI-IBS mice with co-housing treatment.

**Figure 6.**
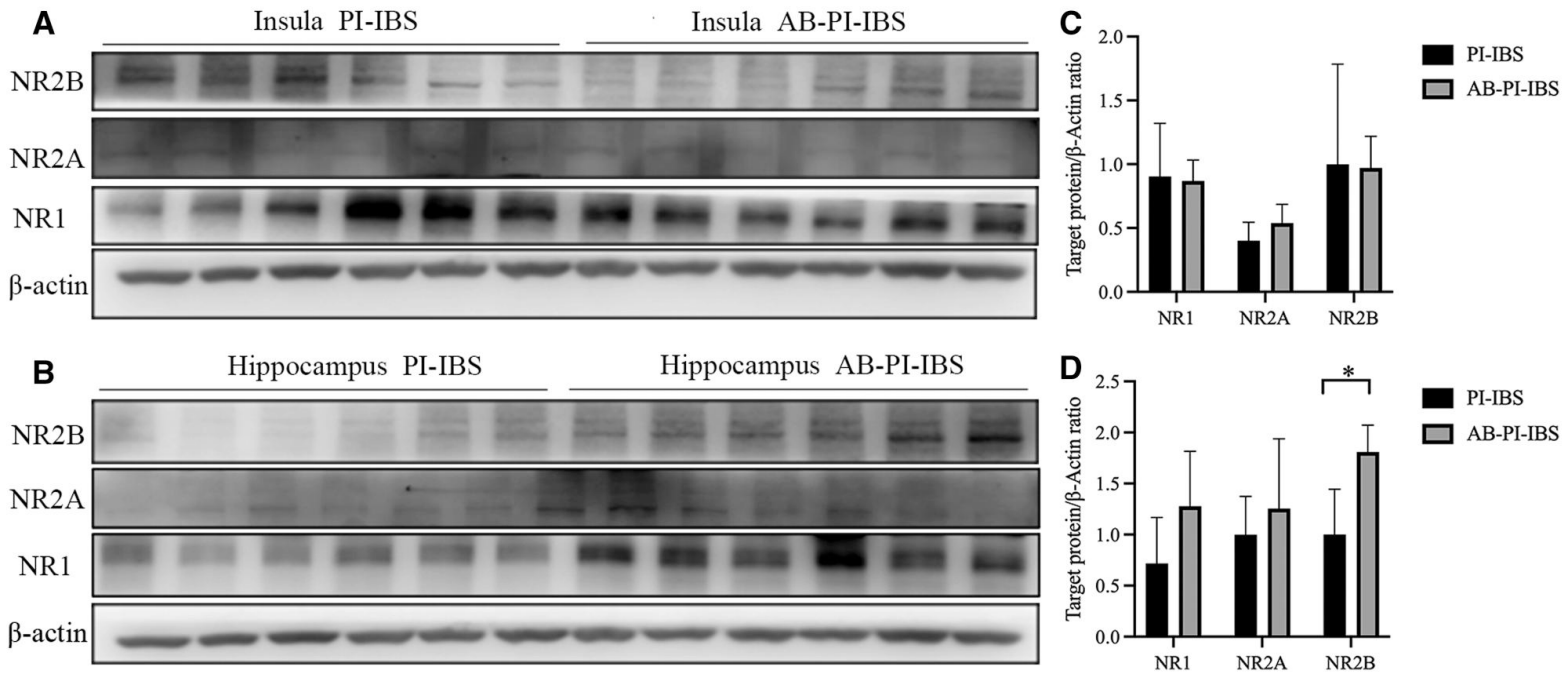
NMDAR expression in PI-IBS mice with antibiotics treatment. (A, C) No difference in NMDAR expression in insula of PI-IBS mice with antibiotics treatment. (B, D) No difference in NMDAR expression in hippocampus of PI-IBS mice with antibiotics treatment. *P < 0.05.

The expression levels of NMDAR subtypes NR1, NR2A, and NR2B were further evaluated. No significant differences were observed in these subtypes between PI-IBS mice and control mice (all *P *> 0.05, Figure 4). However, in PI-IBS mice that experienced co-housing or antibiotics treatment, an increased expression of NR2B was found specifically in the hippocampus after antibiotics therapy (*P *= 0.003, Hedges’ g = 1.144, 95% CI 0.004–2.284, Figures 5 and 6).

### NMDAR/AMPAR eEPSC recording

**Figure 7.**
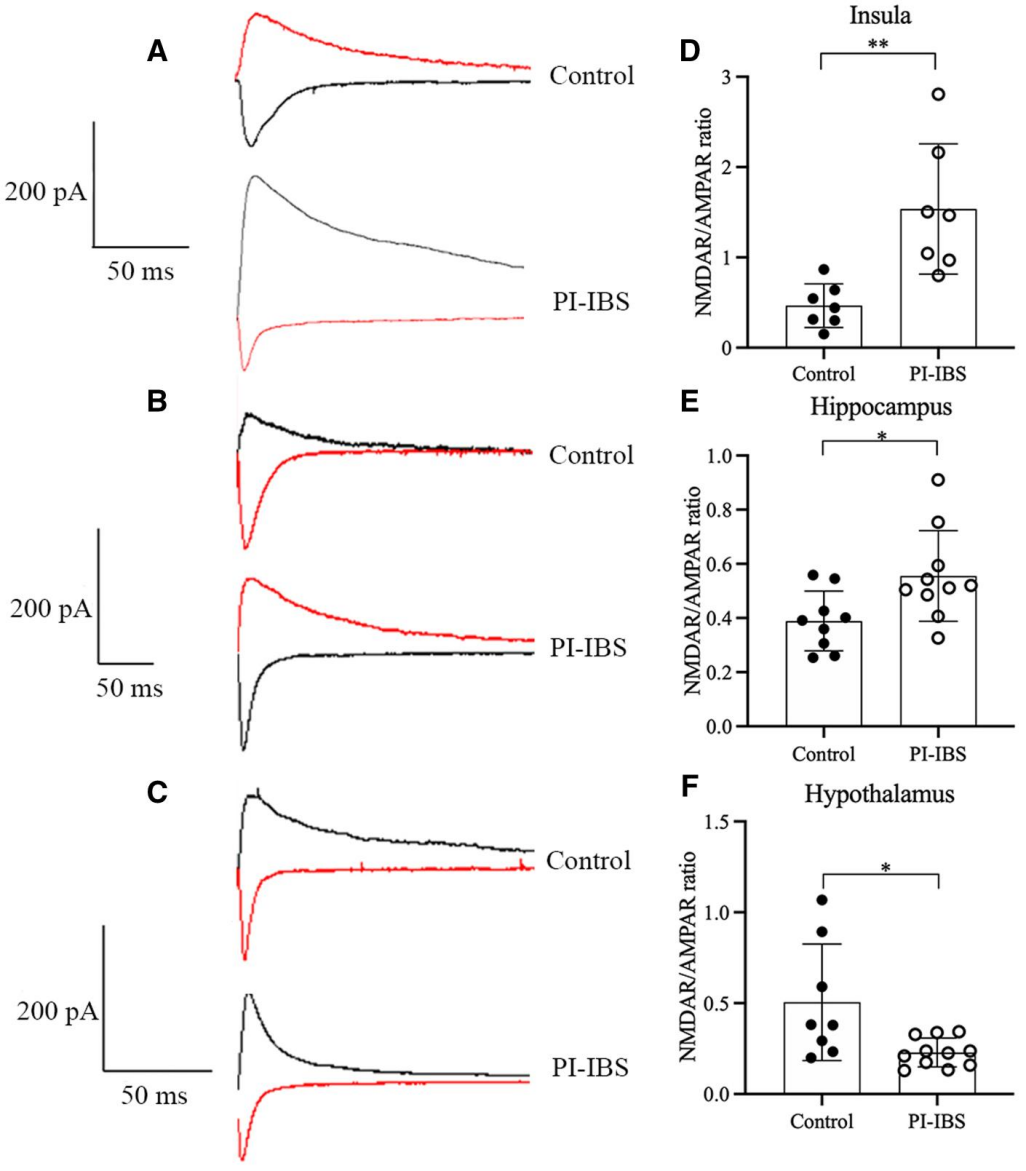
NMDAR/AMPAR-mediated eEPSC in PI-IBS and control mice. (A, D) Increased ratio of NMDAR/AMPAR-mediated eEPSC in the insula of PI-IBS compared with control group. (B, E) Increased ratio of NMDAR/AMPAR-mediated eEPSC in the hippocampus of PI-IBS mice compared with control group. (C, F) Decreased ratio of NMDAR/AMPAR-mediated eEPSC in the hypothalamus of PI-IBS mice compared with control group. *P < 0.05; **P < 0.01.

To evaluate the functional alterations of brain NMDAR in PI-IBS mice, recordings of postsynaptic currents were performed in the insula, hippocampus, and hypothalamus. AMPAR-mediated eEPSC did not show any significant changes between PI-IBS and control groups in the insula, hippocampus, and hypothalamus. However, an increased ratio of NMDAR/AMPAR-mediated eEPSC was observed in the insula (*P *= 0.007, Hedges’ g = 1.993, 95% CI 0.711–3.274) and hippocampus (*P *= 0.022, Hedges’ g = 1.162, 95% CI 0.188–2.135) of PI-IBS mice compared with the control group. On the contrary, a decreased NMDAR/AMPAR ratio was observed in the hypothalamus (*P *= 0.045, Hedges’ g = –1.290, 95% CI –2.289 to –0.291, Figure 7).

**Figure 8.**
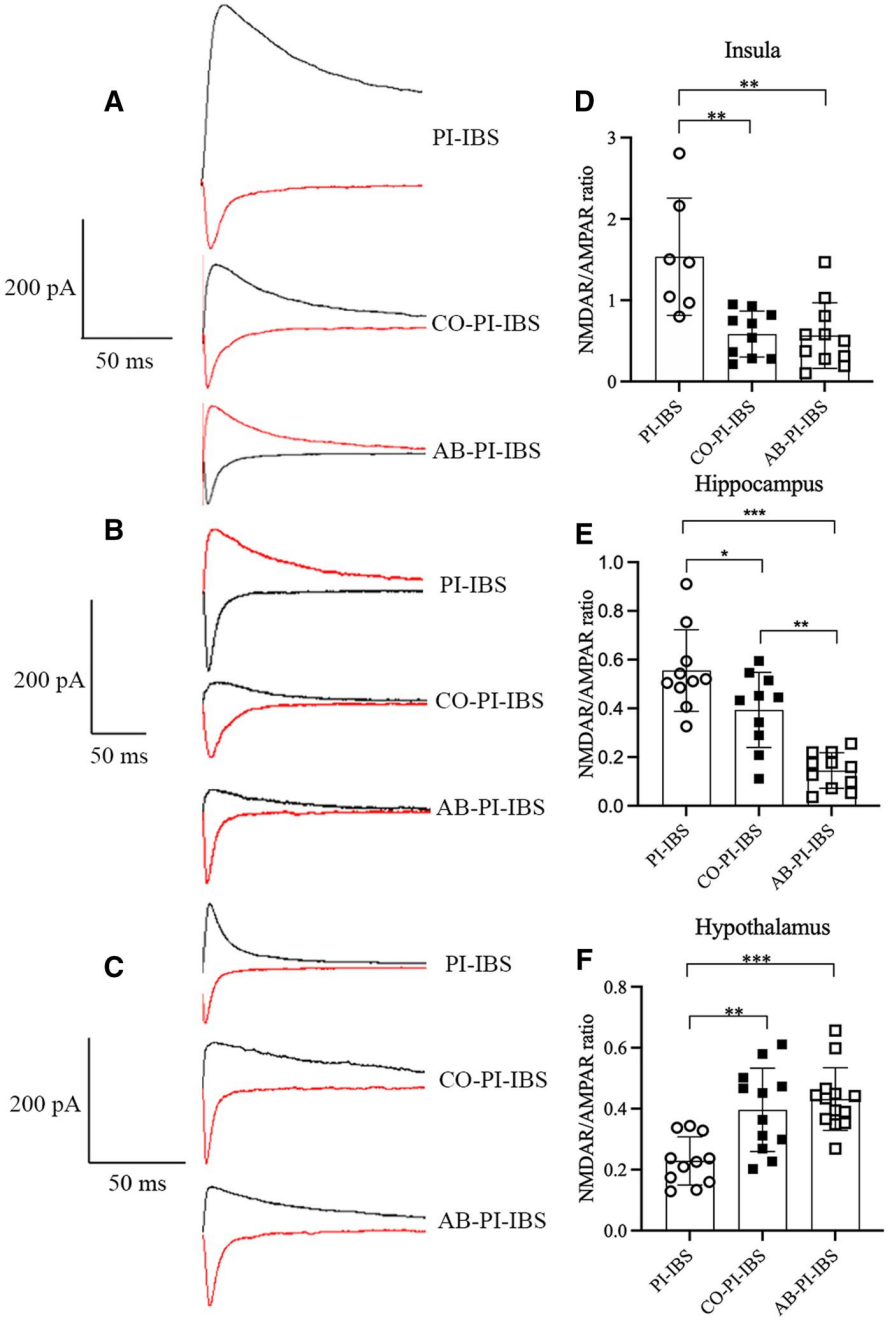
NMDAR/AMPAR-mediated eEPSC in PI-IBS mice with co-housing and antibiotics treatment. (A, D) Decreased ratio of NMDAR/AMPAR-mediated eEPSC in the insula of PI-IBS mice with co-housing and antibiotics treatment. (B, E) Decreased ratio of NMDAR/AMPAR-mediated eEPSC in the hippocampus of PI-IBS mice with co-housing and antibiotics treatment. (C, F) Increased ratio of NMDAR/AMPAR-mediated eEPSC in the hypothalamus of PI-IBS mice with co-housing and antibiotics treatment. *P < 0.05; **P < 0.01.

Following co-housing treatment in PI-IBS mice, AMPAR-mediated eEPSC remained unchanged in the insula, hippocampus, and hypothalamus. However, a decreased ratio of NMDAR/AMPAR-mediated eEPSC was found in the insula (*P *= 0.002, Hedges’ g = –1.882, 95% CI –3.037 to –0.728) and hippocampus (*P *= 0.037, Hedges’ g = –1.007, 95% CI –1.938 to –0.077), whereas an increased ratio of NMDAR/AMPAR-mediated eEPSC was observed in the hypothalamus (*P *= 0.002, Hedges’ g = 1.486, 95% CI 0.562–2.410, Figure 8).

After antibiotics treatment in PI-IBS mice, AMPAR-mediated eEPSC remained unchanged in the insula, hippocampus, and hypothalamus. Similarly to the co-housing treatment, a decreased ratio of NMDAR/AMPAR-mediated eEPSC was observed in the insula (*P *= 0.002, Hedges’ g = –1.781, 95% CI –2.893 to –0.669) and hippocampus (*P *< 0.001, Hedges’ g = –3.239, 95% CI –4.540 to –1.938), whereas an increased ratio was found in the hypothalamus (*P *< 0.001, Hedges’ g = 2.189, 95% CI 1.175–3.202, Figure 8).

## Discussion

In the present study, we identified an enhanced NMDAR/AMPAR-mediated eEPSC ratio within the central nervous system of PI-IBS mice with visceral hypersensitivity. Remarkably, gut microbiota modulation via co-housing and antibiotics treatment normalized the ratio of NMDAR/AMPAR-mediated currents while alleviating visceral hypersensitivity.

NMDAR, critically involved in nociceptive transmission, exhibits dynamic plasticity under both physiological and pathological conditions [11]. Functional NMDAR contains heterometric combinations of the NR1 subunit plus one or more of NR2A-D subunits. Among the NR2 subtypes, NR2A and NR2B subunits are abundantly expressed in various neuronal populations and serve as glutamate-gated ion channels that mediate the transport of Ca^2+^ [25, 26]. Preclinical studies have demonstrated that administration of NMDAR antagonists significantly attenuates the visceromotor response to colorectal distension [27, 28]. Moreover, intrathecal administration of excitatory amino acid receptor agonists evokes spontaneous nociceptive behaviors and hyperalgesia in visceral pain models [29]. Clinically, IBS patients are characterized by visceral hypersensitivity and the upregulation of gut mucosal NMDAR [12].

However, the functional changes in NMDAR within the central nervous system during IBS pathogenesis remains poorly understood. Therefore, our study focused on the NMDAR channels in neuron cells and recorded their dynamic characteristics. Our findings have revealed enhanced NMDAR-mediated currents in the insula and hippocampus of PI-IBS mice, which is consistent with previous reports highlighting increased GluN2B-mediated currents and total NMDAR-mediated responses during chronic visceral pain [30].

Gut microbiota appears to play crucial roles in short-chain fatty acid (SCFA) and some specific amino acids metabolism, highlighting its significance in the microbiota–gut–brain axis. Our prior work has highlighted a significant shift in microbial taxa and microbial-derived SCFAs were associated with disease phenotype in the same experimental model [13]. SCFAs have been found to be able to cross the blood–brain barrier and influence central nervous system activity, including the modulation of microglia activity and cytokine production [31]. Moreover, microbiota also influences central neurotransmitter metabolism [32]. For instance, *Bacteroides vulgatus* and *Campylobacter jejuni* have been found to impact glutamate metabolism and certain lactic acid bacteria can produce glutamate [33, 34]. The activity of microbiota has been associated with specific amino acid concentrations, including D- and L-amino acids, in both the plasma and certain brain regions of mice [35]. These amino acids are key excitatory neurotransmitters for NMDAR [36, 37]. To explore the involvement of microbiota in disease development, approaches such as the elimination of bacteria through the use of broad-spectrum antibiotics or transient co-housing to achieve microbial transfer in a shared environment has been utilized [38, 39]. In our study, we aimed to improve microbial dysbiosis in PI-IBS mice by co-housing them with control mice and mimicking germ-free conditions through antibiotics treatment. Then, we observed a decreased ratio of NMDAR/AMPAR-mediated currents in the insula and hippocampus, accompanied by an improvement in visceral hypersensitivity, while the AMPAR-mediated currents showed no significant changes. These findings suggest a brain–gut–microbiome loop involving microbiota and the NMDAR-mediated activation of an ascending facilitatory system likely contributing to the visceral hypersensitivity observed in PI-IBS. It is conceivable that future research may explore strategies to target specific microbial strains to improve NMDAR activation.

Though our prior work revealed upregulated intestinal NR1 and NR2B subunit expression in PI-IBS mice [13], the current study found no significant alteration in NMDAR subtype expression in the insula and hippocampus between control and PI-IBS mice. Additionally, minimal changes were observed in NMDAR subtypes expression when PI-IBS mice experienced co-housing and antibiotics treatment. Thus, we hypothesize that the NMDAR hyperfunction observed in PI-IBS mice is dependent on enhanced eEPSC rather than the upregulation of receptor expression.

In conclusion, our study identified enhanced NMDAR-mediated eEPSC in the insula and hippocampus of PI-IBS mice with visceral hypersensitivity. Gut microbiota dysbiosis was considered as an initial factor modulating NMDAR hyperfunction. Further investigation of specific microbial species is crucial, as it may provide potential targets for regulating NMDAR function and mitigating visceral hypersensitivity in PI-IBS patients.

## Authors’ contributions

Conception and design of the study: L.D. and N.D. Generation, acquisition of data, assembly, analysis, and/or interpretation of data: L.D., F.C., W.S., Z.F., C.L., Y.Z. Drafting the manuscript: L.D. Approval of the final version of the manuscript: N.D.

## Data Availability

All study-related data are included in the publication or provided as supplementary information.

## References

[1] Enck P , Aziz Q, Barbara G et al Irritable bowel syndrome. Nat Rev Dis Primers 2016;2:16014. 27159638 10.1038/nrdp.2016.14 PMC5001845

[2] Andrews EB , Eaton SC, Hollis KA et al Prevalence and demographics of irritable bowel syndrome: results from a large web-based survey. Aliment Pharmacol Ther 2005;22:935–42. 16268967 10.1111/j.1365-2036.2005.02671.x

[3] Kułak-Bejda A , Bejda G, Waszkiewicz N. Antidepressants for irritable bowel syndrome-A systematic review. Pharmacol Rep 2017;69:1366–79. 29132094 10.1016/j.pharep.2017.05.014

[4] Hoshikawa Y , Hoshino S, Kawami N et al Prevalence of behavioral disorders in patients with vonoprazan-refractory reflux symptoms. J Gastroenterol 2021;56:117–24. 33247348 10.1007/s00535-020-01751-2

[5] Mertz H , Naliboff B, Munakata J et al Altered rectal perception is a biological marker of patients with irritable bowel syndrome. Gastroenterology 1995;109:40–52. 7797041 10.1016/0016-5085(95)90267-8

[6] Collins SM. A role for the gut microbiota in IBS. Nat Rev Gastroenterol Hepatol 2014;11:497–505. 24751910 10.1038/nrgastro.2014.40

[7] Rajilić-Stojanović M , Jonkers DM, Salonen A et al Intestinal microbiota and diet in IBS: causes, consequences, or epiphenomena? Am J Gastroenterol 2015;110:278–87. 25623659 10.1038/ajg.2014.427 PMC4317767

[8] Rhee SH , Pothoulakis C, Mayer EA. Principles and clinical implications of the brain-gut-enteric microbiota axis. Nat Rev Gastroenterol Hepatol 2009;6:306–14. 19404271 10.1038/nrgastro.2009.35 PMC3817714

[9] Nisticò V , Rossi RE, D'Arrigo AM et al Functional neuroimaging in irritable bowel syndrome: a systematic review highlights common brain alterations with functional movement disorders. J Neurogastroenterol Motil 2022;28:185–203. 35189600 10.5056/jnm21079 PMC8978134

[10] Strandwitz P. Neurotransmitter modulation by the gut microbiota. Brain Res 2018;1693:128–33. 29903615 10.1016/j.brainres.2018.03.015 PMC6005194

[11] Traynelis SF , Wollmuth LP, McBain CJ et al Glutamate receptor ion channels: structure, regulation, and function. Pharmacol Rev 2010;62:405–96. 20716669 10.1124/pr.109.002451 PMC2964903

[12] Qi Q , Chen F, Zhang W et al Colonic N-methyl-d-aspartate receptor contributes to visceral hypersensitivity in irritable bowel syndrome. J Gastroenterol Hepatol 2017;32:828–36. 27575648 10.1111/jgh.13588

[13] Cheng F , Fan Z, Lin C et al Effect of altered gut microbiota on visceral hypersensitivity of postinfectious irritable bowel syndrome mice. Eur J Gastroenterol Hepatol 2022;34:1220–30. 36165068 10.1097/MEG.0000000000002441

[14] Guo W , Zou S, Guan Y et al Tyrosine phosphorylation of the NR2B subunit of the NMDA receptor in the spinal cord during the development and maintenance of inflammatory hyperalgesia. J Neurosci 2002;22:6208–17. 12122079 10.1523/JNEUROSCI.22-14-06208.2002 PMC6757905

[15] Zhuo M. Glutamate receptors and persistent pain: targeting forebrain NR2B subunits. Drug Discov Today 2002;7:259–67. 11839523 10.1016/s1359-6446(01)02138-9

[16] Bercik P , Wang L, Verdu EF et al Visceral hyperalgesia and intestinal dysmotility in a mouse model of postinfective gut dysfunction. Gastroenterology 2004;127:179–87. 15236184 10.1053/j.gastro.2004.04.006

[17] Wu M , Li P, An Y et al Phloretin ameliorates dextran sulfate sodium-induced ulcerative colitis in mice by regulating the gut microbiota. Pharmacol Res 2019;150:104489. 31689519 10.1016/j.phrs.2019.104489

[18] Gong S , Lan T, Zeng L et al Gut microbiota mediates diurnal variation of acetaminophen induced acute liver injury in mice. J Hepatol 2018;69:51–9. 29524531 10.1016/j.jhep.2018.02.024 PMC6365016

[19] Jones RC 3rd , Otsuka E, Wagstrom E et al Short-term sensitization of colon mechanoreceptors is associated with long-term hypersensitivity to colon distention in the mouse. Gastroenterology 2007;133:184–94. 17553498 10.1053/j.gastro.2007.04.042

[20] Du L , Long Y, Kim JJ et al Protease Activated Receptor-2 Induces Immune Activation and Visceral Hypersensitivity in Post-infectious Irritable Bowel Syndrome Mice. Dig Dis Sci 2019;64:729–39. 30446929 10.1007/s10620-018-5367-y

[21] Kamp EH , Jones RC 3rd, Tillman SR et al Quantitative assessment and characterization of visceral nociception and hyperalgesia in mice. Am J Physiol Gastrointest Liver Physiol 2003;284:G434–44. 12444012 10.1152/ajpgi.00324.2002

[22] Al-Chaer ED , Kawasaki M, Pasricha PJ. A new model of chronic visceral hypersensitivity in adult rats induced by colon irritation during postnatal development. Gastroenterology 2000;119:1276–85. 11054385 10.1053/gast.2000.19576

[23] Lan C , Sun XN, Zhou XC et al Preinduced intestinal HSP70 improves visceral hypersensitivity and abnormal intestinal motility in PI-IBS mouse model. Asian Pac J Trop Med 2016;9:302–5. 26972407 10.1016/j.apjtm.2016.01.022

[24] Booker SA , Campbell GR, Mysiak KS et al Loss of protohaem IX farnesyltransferase in mature dentate granule cells impairs short-term facilitation at mossy fibre to CA3 pyramidal cell synapses. J Physiol 2017;595:2147–60. 28083896 10.1113/JP273581 PMC5350446

[25] Yi F , Bhattacharya S, Thompson CM et al Functional and pharmacological properties of triheteromeric GluN1/2B/2D NMDA receptors. J Physiol 2019;597:5495–514. 31541561 10.1113/JP278168 PMC6858497

[26] Skeberdis VA , Chevaleyre V, Lau CG et al Protein kinase A regulates calcium permeability of NMDA receptors. Nat Neurosci 2006;9:501–10. 16531999 10.1038/nn1664

[27] McRoberts JA , Coutinho SV, Marvizón JC et al Role of peripheral N-methyl-D-aspartate (NMDA) receptors in visceral nociception in rats. Gastroenterology 2001;120:1737–48. 11375955 10.1053/gast.2001.24848

[28] Coutinho SV , Urban MO, Gebhart GF. The role of CNS NMDA receptors and nitric oxide in visceral hyperalgesia. Eur J Pharmacol 2001;429:319–25. 11698052 10.1016/s0014-2999(01)01331-0

[29] Rice ASC , McMahon SB. Pre-emptive intrathecal administration of an NMDA receptor antagonist (AP-5) prevents hyper-reflexia in a model of persistent visceral pain. Pain 1994;57:335–40. 7936711 10.1016/0304-3959(94)90009-4

[30] Liu SB , Wang XS, Yue J et al Cyclic AMP-dependent positive feedback signaling pathways in the cortex contributes to visceral pain. J Neurochem 2020;153:252–63. 31665810 10.1111/jnc.14903

[31] Wenzel TJ , Gates EJ, Ranger AL et al Short-chain fatty acids (SCFAs) alone or in combination regulate select immune functions of microglia-like cells. Mol Cell Neurosci 2020;105:103493. 32333962 10.1016/j.mcn.2020.103493

[32] Ahmed H , Leyrolle Q, Koistinen V et al Microbiota-derived metabolites as drivers of gut-brain communication. Gut Microbes 2022;14:2102878. 35903003 10.1080/19490976.2022.2102878 PMC9341364

[33] Chang CH , Lin CH, Lane HY. d-glutamate and Gut Microbiota in Alzheimer's Disease. Int J Mol Sci 2020;21:2676. 32290475 10.3390/ijms21082676 PMC7215955

[34] Zareian M , Ebrahimpour A, Bakar FA et al A glutamic acid-producing lactic acid bacteria isolated from Malaysian fermented foods. Int J Mol Sci 2012;13:5482–97. 22754309 10.3390/ijms13055482 PMC3382744

[35] Kawase T , Nagasawa M, Ikeda H et al Gut microbiota of mice putatively modifies amino acid metabolism in the host brain. Br J Nutr 2017;117:775–83. 28393748 10.1017/S0007114517000678

[36] Niciu MJ , Kelmendi B, Sanacora G. Overview of glutamatergic neurotransmission in the nervous system. Pharmacol Biochem Behav 2012;100:656–64. 21889952 10.1016/j.pbb.2011.08.008 PMC3253893

[37] Dicks LMT. Gut Bacteria and Neurotransmitters. Microorganisms 2022;10:1838. 36144440 10.3390/microorganisms10091838 PMC9504309

[38] Murray E , Butcher J, May Kearns M et al Effects of pair-housing pubertal and adult male and female mice on LPS-induced age-dependent immune responses: A potential role for the gut microbiota. Brain Behav Immun 2023;110:297–309. 36914014 10.1016/j.bbi.2023.03.009

[39] Surana NK , Kasper DL. Moving beyond microbiome-wide associations to causal microbe identification. Nature 2017;552:244–7. 29211710 10.1038/nature25019 PMC5730484

